# Role of inflammatory markers in Takayasu arteritis disease monitoring

**DOI:** 10.1186/1471-2377-14-62

**Published:** 2014-03-28

**Authors:** Timothy E O’Connor, Haley E Carpenter, Sharatchandra Bidari, Michael F Waters, Vishnumurthy Shushrutha Hedna

**Affiliations:** 1 College of Medicine, University of Florida, Gainesville, FL, USA; 2 Department of Neuroscience, University of Florida, Gainesville, FL, USA; 3 Department of Neurology, University of Florida, Room L3-100, McKnight Brain Institute 1149 Newell Drive, Gainesville, FL 32611, USA; 4 Department of Radiology, University of Florida, Gainesville, FL, USA

**Keywords:** Takayasu arteritis, ESR, CRP, Compensatory circulation, Subclavian steal

## Abstract

**Background:**

Takayasu arteritis (TA) is an idiopathic large-vessel vasculitis that can result in significant morbidity and mortality secondary to progressive stenosis and occlusion. Monitoring disease progression is crucial to preventing relapse, but is often complicated by the lack of clinical symptoms in the setting of active disease. Although acute phase reactants such as ESR and CRP are generally used as an indicator of inflammation and disease activity, mounting evidence suggests that these markers cannot reliably distinguish active from inactive TA.

**Case presentation:**

We report a 24-year-old Hispanic female with a 5-year history of TA who presented with stroke-like symptoms and evidence of left MCA occlusion on imaging, despite a history of decreasing inflammatory markers. CTA revealed complete occlusion of the left common carotid artery, left subclavian, and left MCA from their origins. It also revealed a striking compensatory circulation supplying the left anterior circulation as well as the left subclavian as a response to progressive stenosis.

**Conclusion:**

Monitoring ESR and CRP levels alone may not be a reliable method to evaluate disease progression in patients with TA, and should be taken in context with both patient’s clinical picture and the imaging. We recommend that serial imaging be performed regularly in the setting of active disease to monitor progression and allow for immediate therapy in response to evidence of disease advancement, with a relaxation of the imaging interval once the disease is presumed inactive.

## Background

Takayasu arteritis (TA) is an idiopathic large vessel vasculitis that primarily affects the aorta and its main branches. Although the prevalence and clinical outcome of TA varies globally, the annual incidence of TA in the United States is reportedly 2.6 per million with a 5-year survival rate as high as 94% [1,2]. The disease predominantly affects women and typically presents during the second to third decade of life [3]. Signs and symptoms of TA are diverse and reflect both the stage of the disease and the affected vasculature. Early stage TA can present with nonspecific symptoms such as fatigue, weight loss, and low grade fever, but as the disease progresses it can manifest as vascular bruits, claudication, retinopathy, and ischemia due to arterial occlusion [4]. The evolution of these symptoms is due to progressing vascular lesions secondary to inflammatory processes [5].

TA is generally monitored closely because relapses are often unpredictable and dangerous. Inflammatory markers such as erythrocyte sedimentation rate (ESR) and C - reactive protein (CRP) are commonly used to monitor disease progression during remission due to their noninvasive nature and affordable cost, while the radiation diagnostic technique CT angiography (CTA) is reserved for patients presenting with active disease. Despite these monitoring techniques, assessment of disease activity and progression in TA remains a challenge [6]. However, relying on the inflammatory marker ESR to distinguish active from inactive TA yields only a 72% sensitivity and a 56% specificity predictor value [7]. As a result, relying on these markers during asymptomatic periods can potentially lead to the false assumption that the patient is in remission while there is ongoing active fibrosis and progressive occlusion. In fact, one study found histologically active disease in 44% of cardiac biopsy specimens and angiographic evidence of progression in 61% of patients previously believed to be in remission [7]. As a result, mounting evidence suggests it may be necessary to use more invasive imaging to accurately determine active disease.

We present a patient who suffered an ischemic stroke in spite of improving inflammatory markers suggestive of disease remission. This patient’s clinical course supports the hypothesis that inflammatory markers alone may not be sufficient to track the progression of TA, and reaffirms the need to monitor disease progression with more extensive screening. In addition, this patient demonstrated a remarkable capacity via collaterals to compensate in the setting of progressive occlusion. In response to active disease, utilization of vascular reserve through collateral circulation eventually resulted in retrograde flow through the vertebral artery to perfuse tissue distal to severe stenosis of the common carotid artery (CCA).

## Case presentation

**Figure 1:**
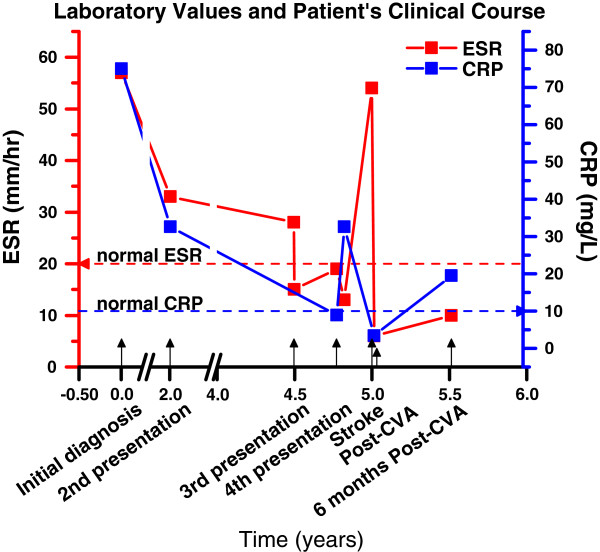
ESR and CRP values over timespan of treatment.

A 24 year-old woman with Takayasu’s arteritis (TA) and a recent history of progressive diffuse headaches presented to an outside hospital with altered mental status following loss of consciousness. Five years previously the patient was diagnosed with anti-myeloperoxidase (MPO) and p-ANCA vasculitis with an ESR of 57 mm/hr and a CRP of 75 mg/L (Figure 1, initial diagnosis). The patient was prescribed cellcept and a prednisone taper, but was lost to follow up until two years later when she developed hand numbness, headaches, and postprandial abdominal pain. CTA revealed 90% stenosis of the superior mesenteric artery, and ESR and CRP were elevated at 33 mm/hr and 32 mg/L, respectively (Figure 1, 2^nd^ presentation). The patient was treated with cellcept and high-dose prednisone but was again lost to follow up.

At the age of 23 the patient presented to the emergency department with postprandial abdominal pain, and CTA indicated progression of her disease with luminal narrowing due to intimal thickening of the aortic arch, descending aorta, and left subclavian artery that was characteristic of large vessel disease consistent with Takayasu’s arteritis. Her ESR and CRP were 28 mm/hr and 7.1 mg/L, respectively, and the following day, her ESR was 15 mm/hr (Figure 1, 3^rd^ presentation). Imuran 50 mg/d was started with plans to titrate the dose; however she discontinued her treatment after 3 weeks due to persistent nausea and vomiting.

**Figure 2:**
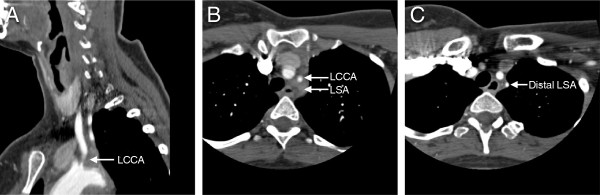
**CTAs three months before CVA.** Oblique lateral MPR view CT angiogram of the neck showing a thrombosed left common carotid artery (LCCA) with a high-grade stenosis at the origin of the common carotid, 1 cm in length **(A)**. Transverse CTA just above the origin of the great vessels revealing complete occlusion of the left proximal subclavian artery (LSA) **(B)**, with flow to the distal left subclavian artery provided by the left vertebral artery, indicative of subclavian steal **(C)**.

Four months later the patient complained of progressive, persistent, diffuse pounding headaches that were associated with dizziness and blurry vision, and she was admitted to the hospital for further evaluation. At that time she was not on any immunosuppressant therapy. ESR was within normal limits at 19 mm/hr and CRP was only mildly elevated at 8.9 mg/L (Figure 1, 4^th^ presentation). But CTA of the head, neck, chest, abdomen, and pelvis demonstrated further progression of her disease with circumferential thickening of the aortic arch and significant narrowing of the proximal descending thoracic aorta, along with a severe stenosis of the left common carotid artery at its origin, 1 centimeter in length (Figure 2A). There was complete occlusion of the left proximal subclavian artery at its origin from the aortic arch, with flow in the distal left subclavian artery provided by the left vertebral artery, suggestive of subclavian steal (Figure 2B), and with reconstitution of flow at the bifurcation of the vertebral artery suggesting collateral flow was arising from the posterior circulation. Carotid ultrasound indicated the left vertebral artery was patent with retrograde flow. Imuran was stopped and cellcept was restarted with a 60 mg prednisone taper; however, she refused pulse steroids due to concerns weight gain and also refused cytoxan again. Over the next several weeks the patient continued to experience worsening headaches unrelieved by over the counter pain medications. She also became noncompliant with cellcept.

One month later, the patient presented to an outside hospital with right sided weakness and speech difficultly. After CT ruled out hemorrhage, recombinant tissue plasminogen activator (t-PA) was administered within the therapeutic window and the patient was transferred to our hospital for further work up. On admission, the patient was limited to following one step commands. Neurologic exam demonstrated anomia, dysarthria, a right lower facial droop, and severe weakness on the right upper and lower extremity. The strength in left upper and lower extremities was intact, and the rest of the physical exam was unremarkable.

**Figure 3:**
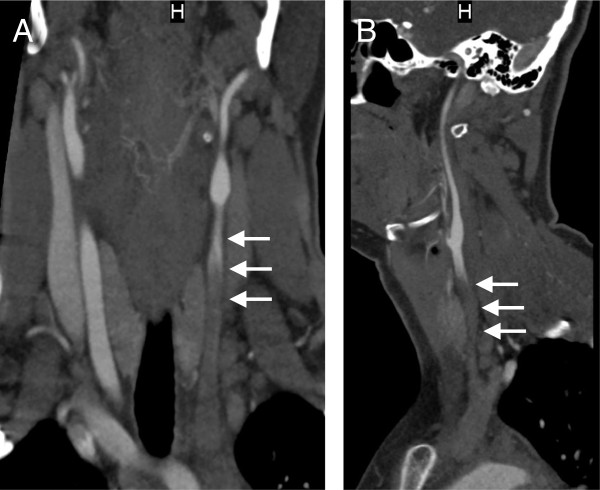
**CTA of the neck immediately following CVA.** Anterior **(A)** and lateral **(B)** views of a CT angiogram of the neck demonstrating thrombosis of the proximal and mid left common carotid artery (arrows).

**Figure 4:**
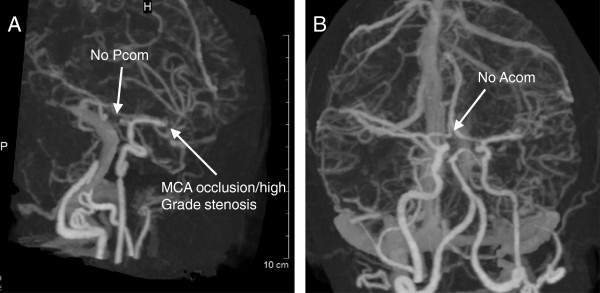
**Subtracted 3D CT angiogram following CVA.** Oblique view showing high-grade stenosis of left MCA with M2 M3 branches opacified by retrograde flow through leptomeningeal collaterals **(A)**. Neither posterior communicating artery **(A)** nor anterior communicating artery **(B)** visible upon imaging in oblique and AP views, respectively.

**Figure 5:**
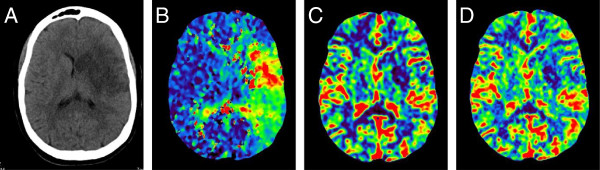
**Brain CT imaging post CVA.** Brain CT showing completed infarct in left middle cerebral artery territory affecting the left basal ganglia and left insular cortex. Cytotoxic edema present **(A)**. CT perfusion shows increased time to peak **(B)**, reduced cerebral blood volume **(C)** and reduced cerebral blood flow **(D)** in the core left MCA territory.

CTA of the head and neck demonstrated mural thickening of the aortic arch that was consistent with previous images. There was also complete occlusion of the left common carotid artery at its origin (Figure 3) and left internal carotid artery, with collateral flow arising from the right anterior circulation via the circle of Willis and leptomeningeal collaterals. In addition, there is complete occlusion of a short segment of the MCA, just past its bifurcation (Figure 4A) and evidence of flow through the anterior and posterior communicating arteries is absent upon imaging (Figure 4). These findings would explain the CT perfusion abnormalities of delay in transit time with decreased cerebral blood flow and cerebral blood volume, consistent with the left MCA infarct, including the left basal ganglia (Figure 5).

Initially, the patient’s ESR was elevated at 54 mm/hr (Figure 1, stroke). The patient was started on pulse steroids the day following her stroke. On the fifth day of admission repeat ESR was 6 mm/hr and CRP was within normal limits at 3.4 mg/L (Figure 1, Post-CVA). Strength in the right upper and lower extremity improved while facial droop and speech deficit persisted. After 8 days the patient was discharged to an inpatient rehabilitation facility, where she continued to improve and can now walk unassisted and perform all of her activities of daily living.

**Figure 6:**
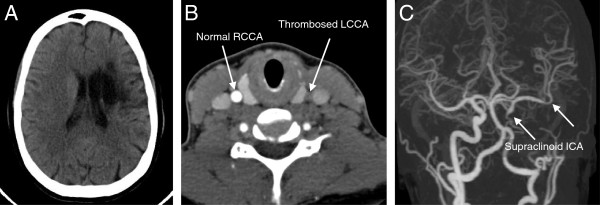
**Imaging from 6 months following initial acute CVA.** Brain CT showing encephalomalacia related to the previous infarct of the left basal ganglia and left insular cortex (left MCA territory). **(A)** Transverse slice through neck showing complete occlusion of left common carotid artery **(B)**. Subtracted 3D CT angiogram showing a complete lack of flow in left internal carotid artery as well as a recanalization of the previous area of left MCA thrombosis **(C)**.

Six months following the patient’s cerebrovascular attack (CVA), the patient’s ESR returned to the normal range at 10 mm/hr and her CRP value remained elevated at 19.5 mg/L (Figure 1, 6 months post-CVA). CTA of the head and neck showed encephalomalacia related to the previous infarct, complete occlusion of the left common carotid artery, and a striking complete lack of flow in the left internal carotid artery (Figure 6).

## Discussion

We presented a patient who experienced progressive vascular lesions due to TA culminating in an ischemic stroke despite normalizing levels of inflammatory markers. TA is a large-vessel pan-arteritis affecting predominantly the aorta and its major branches. Its etiology is unknown, but its pathogenesis is suggestive of an autoimmune process and is likely multifactorial [5]. The pathophysiology of this disease can be divided into early, intermediate and chronic stages [3]. Early in the disease process, lymphocytes infiltrate the adventitia through activated vasa-vasorum endothelial cells. The lymphocytes are stimulated by circulating cytokines to produce matrix metalloproteinases (MMP), leading to the destruction of the elastic fibers in the arterial wall. Increased adventitial neovascularization and up-regulation of adhesion molecules results in increased recruitment of inflammatory cells. In the intermediate stage, there is secondary deposition of mucopolysaccharides and fibroblasts and smooth muscle cell proliferation, encouraged by TNF-alpha. Eventually the intima becomes hypertrophied due to fibrocellular thickening. The pathological changes occurring in all layers lead to the narrowing of the vascular lumen, which result in stenosis and occlusion. In the chronic phase, inflammatory lesions progress to scars and the vessels become fibrotic and calcified [8]. When fibrosis is insufficient, as when lesions progress rapidly, the arterial wall is thinned and dilation and aneurysms occur. In addition to the primary lesions of TA that occur in the elastic arteries, affecting both the media and adventitia through the vasa-vasorum, peripheral branches of affected arteries are disrupted as well. In these peripheral branches, intimal thickening occurs in the absence of associated changes in the media and adventitia, leading to occlusion and secondary ischemic lesions to the kidneys, heart, and brain [9]. The spread of vascular lesions in TA is thought to develop systemically rather than from adjacent vascular beds [5].

Although our patient was managed using cellcept and prednisone, her noncompliance made it difficult to maintain remission. Traditional therapy for TA consists of corticosteroids, with immunosuppressants and cytotoxic agents prescribed in treatment-resistant and refractory cases. Reports indicate that half of all patients who achieve remission through treatment will eventually relapse, and one fourth of patients are unable to achieve remission with either glucocorticoids or glucocorticoids in combination with cytotoxic agents [7]. Alternative therapies that have been used with success in refractory cases of TA include infliximab, tocilizumab, rituximab, tacrolimus, methotrexate, azathioprine, cyclophosphamide, mycophenolate mofetil, and leflunomide [10]–[14]. Given the high probability of relapse and potential disastrous consequences, it is necessary to frequently and reliably monitor progression.

In every phase of TA management, from the initial diagnosis to the monitoring of quiescent periods, flares and remission, clinical examination, biomarker data, and imaging are all critical components TA evaluation. None of these assessment techniques can be used in isolation to sufficiently evaluate disease state [15].

For the clinical assessment of TA, several indexes have been developed. For TA diagnosis, the 1990 American College of Rheumatology (ACR) criteria for TA classification was developed. This index contains of six criteria: 1) Age of onset <40 years; 2) Claudication of extremities; 3) Decreased brachial artery pulse; 4) A systolic blood pressure difference of >10 mm Hg between arms; 5) A bruit of the subclavian arteries or aorta; and 6) An arteriogram abnormality. If three or more of these criteria are met, a TA diagnosis can be made with a sensitivity of 90.5% and a specificity of 97.8% [16]. TA disease activity can be assessed with the National Institutes of Health (NIH) criteria for active disease, with active disease defined as the new onset or worsening of two of the following four criteria: 1) Systemic features, such as fever and malaise, with no other cause identified; 2) Elevated erythrocyte sedimentation rate; 3) features of vascular ischemia or inflammation (claudication, diminished or absent pulses, bruit, vascular pain, asymmetric blood pressure); and 4) Typical angiographic features [7]. The Disease Extent Index-Takayasu (DEI-Tak) is an index consisting of 71 items designed for the follow-up of TA based solely on clinical findings, eliminating the requirement of imaging. Though time-consuming to administer, the DEI-Tak shows good agreement with the NIH criteria [17]. The Indian Takayasu’s Arteritis Score (ITAS) was developed from the DEI-Tak and modified to optimize inter-rater reliability and reflection of disease activity. The ITAS consists of 44 items, 33 of which pertain to the cardiovascular system, and 7 of which are weighted more heavily. The ITAS is sensitive to effective medical interventions, and a high ITAS score denotes poor control of disease activity [18].

This patient’s case is unique because inflammatory markers were decreasing over several years despite active disease and progressive lesions. Acute-phase reactants such as ESR and CRP are commonly used to monitor disease progression in TA. Despite their usage, neither ESR nor CRP can distinguish active from inactive disease with the necessary clinical accuracy [19,20]. ESR has a sensitivity and specificity for active TA of 72% and 56%, while CRP has a sensitivity of 71.4% and specificity up to 100% for active disease [7,21] (Table 1). However, TA can relapse and active disease can persist in the absence of elevated CRP and ESR [21]. Neither ESR nor CRP correlate with MR findings of vascular edema identified with electrocardiogram-gated edema-weighted MR [22]. In addition, both ESR and CRP are suppressed by factors other than disease remission. ESR can be influenced by extraneous factors such as medication usage and blood viscosity and CRP exhibits nonspecific elevation in response to tissue inflammation and infection. Additionally, CRP is produced in the liver in response to the circulating cytokine IL-6, rather than at the site of inflammation, which may account for its low sensitivity [7,21,23]. Relying on these inflammatory markers alone to monitor disease progression can have severe and even lethal consequences for patients [8,10]. Therefore, ESR and CRP should be viewed in the context of the patient’s clinical course and imaging.

**Table 1:** Efficacy of serological markers and imaging modalities in the diagnosis of TA

**Modality**	**Sensitivity (%)**	**Specificity (%)**	**Citation**
ESR	72*	56*	Kerr, Hallahan, Giordano, et al., 1994^†,‡^[7]
CRP	71.4*	100*	Ishihara, Haraguchi, Tezuka, et al., 2012^†,§,¶^[21]
FDG-PET	92*	100*	Webb, Chambers, AL-Nahhas, et al., 2004^†,‡,§^[34]
MRA	100*	100*	Yamada, Nakagawa, Himeno, et al., 2000^‡ ^[31]
CTA	95*	100*	Yamada, Nakagawa, Himeno, et al., 1998^‡^[27]
PTX-3	82.1-89**	87-94.1**	Ishihara, Haraguchi, Tezuka, et al., 2012^†,§,¶^, [21] Dagna, Salvo, Tiraboschi, et al., 2011^†,‡,§^[23]

*In diagnosing TA.

**In distinguishing active from inactive disease.

TA diagnosis or disease activity state determined by;

^†^Clinical assessment.

^‡^Typical findings on conventional angiography.

^§^Patients met 1990 American College of Rheumatology criteria for TA diagnosis.

^¶^MRA.

The unreliability of ESR and CRP has prompted the search for a more reliable serological marker of disease activity. Many biomarker candidates have been identified. Park et al. examined the serum profiles of inflammatory cytokines and found that TNF-alpha, IL-6 and IL-18 were elevated in TA, and of these, IL-18 correlated best with remission [24]. De Souza et al. found that higher homocysteine levels in TA is a risk factor for cardiovascular events [25]. In evaluating serum ghrelin and leptin levels as potential biomarkers for TA, Yilmaz et al. found that ghrelin levels were negatively correlated with disease activity and that the leptin/ghrelin ratio was higher in TA [26]. Regulated and normal T cell-expressed and secreted (RANTES) levels are increased in active TA and correlate with disease activity [5]. Dagna et al. found that Pentraxin-3 (PTX-3) levels are positively correlated with disease state and determined PTX-3 to be more predictive of TA disease state than ESR or CRP, with a sensitivity of 89% and specificity of 87%. An additional benefit of PTX-3 over conventional markers is that PTX-3 did not show nonspecific elevation in healthy controls or in response to infection [23]. Though all require further validation, these serological markers show promise as future biomarkers for TA.

In addition to biomarkers, imaging is indispensable for the diagnosis and monitoring of TA. Imaging methods useful for TA include digital subtraction angiography (DSA), computed tomography angiography (CTA), magnetic resonance imaging (MRI), ultrasonography, and positron emission tomography with radiolabelled glucose (FDG-PET). Angiography can be used for both screening and treatment. It has a high sensitivity and specificity for TA diagnosis, a short study time, is minimally invasive, and allows for easy comparison of studies performed throughout disease progression. In comparison to standard angiography, CTA has a high sensitivity and specificity for assessing stenotic lesions, 93% and 98%, respectively, with an overall sensitivity and specificity of 95% and 100% in diagnosis [27]. CTA allows evaluation of the vessel wall and lumen in the aorta and large vessels and can provide information concerning end organ ischemia. However, it is costly, exposes the patient to higher radiation than other imaging modalities, and is impractical for frequent monitoring [8,28].

MRI is noninvasive, lacks radiation, and can detect anatomic and pathophysiologic changes such as vascular lesions, sites of inflammation, and wall thickening [26]. T2 imaging can visualize vessel edema, a sign of inflammation, and a specialized T2 weighted technique, short tau inversion recovery (STIR), is ideal for imaging soft tissue inflammation [22,29]. MR angiography (MRA) with gadolinium contrast facilitates visualization of pathological wall enhancement and has demonstrated a sensitivity and specificity of up to 100% in diagnosing TA [30,31]. However, MRI is time consuming, expensive, can be affected by movement artifact, cannot be performed in patients with ferromagnetic implants, and may require 3 Tesla strength in order to visualize smaller vessels [8].

Ultrasonography (US) is non-invasive, cheap, painless, and avoids ionizing radiation, making it excellent tool for frequent monitoring of TA. It can be used to identify the presence of stenosis, estimate blood flow, and assess vessel anatomy, the lumen, and vessel wall alterations. Additionally, US has a high resolution of approximately 0.1 mm, providing 10x the resolution of MRI [32,33].

FDG-PET can assess metabolic activity of the vascular wall, highlighting regions of inflammation. As inflammation begins before morphological changes in the arterial wall manifest, FDG-PET can facilitate earlier diagnosis. FDG-PET has shown a sensitivity of 92% and a specificity of 100% in diagnosing TA and is more sensitive than MRA in detecting vascular involvement in early TA [34,35]. PET findings of inflammation normalize after treatment with immunosuppressants, mirroring clinical improvement, making PET a reliable indicator of disease activity [32]. FDG-PET is useful for screening patients with systemic diseases and diagnosing TA [36]. However, it is expensive, not widely available, exposes the patient to radiation, is limited to vessels greater than 4 mm in diameter, and cannot be used to examine the vessel wall [28,32].

In our patient, it is possible that complications from her disease could have been prevented if serial imaging allowed for treatment to be implemented before manifestations occurred. However, the literature guiding how TA should be managed and how often imaging should be performed in these patients is limited, lacking placebo-controlled, randomized controlled trials and consisting primarily of case series, open studies, and expert opinion [15]. Additionally, local considerations, such as technique availability and provider expertise, will also impact which imaging modality is implemented, further complicating the concept of a universal imaging algorithm for TA [28]. Three case reports provide insight on feasible imaging intervals. In the first case, improvement in vascular lesions on CTA was seen within 6 months of initial presentation with an ischemic stroke [3]. The second report was an instance of TA causing pulmonary stenosis and occlusion. Improvement of lesions was shown on four imaging modalities, gadolinium-enhanced MRI, two dimensional transesophageal echocardiography (TEE), pulmonary angiography, and FDG-PET. Remarkably, improvements were seen after only 6 weeks of steroid therapy [37]. In a third report, TA diagnosis was made with FDG-PET, which showed F-18 FDG uptake in the aorta, subclavian, and brachiocephalic arteries. Following two months of successful immunosuppressant therapy, with remission of clinical symptoms, the patient was again imaged with FDG-PET, revealing a dramatic decrease in F-18 FDG uptake in the affected vessels [38]. Serial imaging is important for monitoring TA patients both for remission and relapse, to gauge the effectiveness of current therapies and to detect subclinical progression of vascular lesions. We recommend conservative imaging intervals for both of these periods, with more frequent imaging during active disease and regular imaging even in the absence of clinical symptoms during remission.

Another remarkable feature of this case was the labyrinthine supply of cerebral blood flow, with collateral and retrograde flow compensating for severe stenosis and eventual complete occlusion of several vessels. In our patient, both her left common carotid artery and left subclavian artery progressed from severe stenosis to complete occlusion. Despite complete occlusion at its origin, the left common carotid was patent. CTA imaging revealed the occipital artery was the predominant collateral supply to the left anterior circulation, supplying retrograde flow through the external carotid. Additionally, Transcranial Doppler revealed stenosis of her left subclavian artery was compensated by retrograde flow through her left vertebral artery. It has been reported that the slow progression of stenosis can allow for the development of collateral circulation, delaying or minimizing symptoms of vascular compromise [4]. Our patient’s compensatory circulation was protective against left hemisphere ischemia and delayed the onset of neurological symptoms. This case demonstrates the incredible compensatory capacity of these vessels in the setting of ongoing stenosis.

This patient suffered an ischemic stroke due to the progressive occlusion of her cerebral arteries secondary to her TA. In some cases, when the threat from stenosis becomes severe, endovascular or surgical revascularization procedures may be required. These procedures should be reserved for the inactive phase of the disease, and for stents, disease flare ups can cause re-occlusion of both the affected artery and the stent. Balloon angioplasty and stent graft replacements can be used in the setting of short-segment arterial stenosis, whereas long-segment stenosis requires a complete surgical bypass [15]. It is possible that the current patient could have benefitted from revascularization procedures, especially around the time of her fourth presentation, when critical stenosis of several extracranial large vessels was becoming apparent and both her ESR and CRP were within normal limits.

## Conclusions

This case suggests that inflammatory markers alone are not sufficient to monitor disease activity, highlighting the need for frequent follow up imaging. We recommend that serial imaging be performed regularly in the setting of active disease until patient demonstrates evidence of remission, with an extended imaging interval in the context of inactive disease. This case is also serves as a reminder of the importance of regular visits and compliance in TA management. This patient had extended periods between visits, presenting only when symptoms reemerged, and frequently became noncompliant with medication. It is likely that her disease course would have been less severe with more robust patient education, continued communication with providers, regular follow up, and medication compliance.

These time intervals provide a means to assess treatment efficacy and allow for immediate intervention in response to disease exacerbation. Furthermore, this case provides a striking example of how collateral and retrograde circulation can compensate for the progressive stenosis associated with TA.

## Consent

Written informed consent was obtained from the patient for publication of this case report and any accompanying images. A copy of the written consent is available for review by the Editor-in-Chief of this journal.

## Abbreviations

ESR: Erythrocyte sedimentation rate; CRP: C-reactive protein; MCA: Middle cerebral artery; CTA: CT angiogram; MRA: Magnetic resonance angiogram; CCA: Common carotid artery; CVA: Cerebrovascular attack; MMP: Matrix metalloproteinase; TNF-alpha: Tumor necrosis factor alpha; PTX: Pentraxin; TEE: Transeophageal echocardiogram.

## Competing interests

The authors declare that they have no competing interests.

## Authors’ contributions

Study concept and design: TEO, HEC, VSH. Acquisition of data: TEO, HEC. Analysis and interpretation of data: TEO, HEC, VSH. Drafting and critical revision of manuscript: TEO, HEC, VSH. Statistical analysis: TEO, HEC, VSH. Administrative, technical, and material support: SB, MFW, VSH. Study supervision: SB, MFW, VSH. All authors read and approved the final manuscript.

## Pre-publication history

The pre-publication history for this paper can be accessed here:

http://www.biomedcentral.com/1471-2377/14/62/prepub
